# The Preparation, Determination of a Flexible Complex Liposome Co-Loaded with Cabazitaxel and β-Elemene, and Animal Pharmacodynamics on Paclitaxel-Resistant Lung Adenocarcinoma

**DOI:** 10.3390/molecules24091697

**Published:** 2019-04-30

**Authors:** Yi-Ying Zeng, Yi-Jun Zeng, Na-Na Zhang, Chen-Xi Li, Tian Xie, Zhao-Wu Zeng

**Affiliations:** 1 Institute of Chinese Materia Medica, Shanghai University of Traditional Chinese Medicine, 1200 Cailun Road, Shanghai 201203, China; zengyiying2008@163.com; 2 Holistic Integrative Pharmacy Institutes, Hangzhou Normal University, 1378 Wenyi Road, Hangzhou 311121, China; zy0508j@163.com (Y.-J.Z.); nanazhang2019@126.com (N.-N.Z.); chenxichenhaochen@163.com (C.-X.L.); 3 Key Laboratory of Elemene Class Anti-Cancer Chinese Medicine of Zhejiang Province, Hangzhou 311121, China; 4 Engineering Laboratory of Development and Application of Traditional Chinese Medicine from Zhejiang Province, Hangzhou 311121, China

**Keywords:** cabazitaxel, β-elemene, liposome, paclitaxel resistance, lung adenocarcinoma

## Abstract

Paclitaxel is highly effective at killing many malignant tumors; however, the development of drug resistance is common in clinical applications. The issue of overcoming paclitaxel resistance is a difficult challenge at present. In this study, we developed nano drugs to treat paclitaxel-resistant lung adenocarcinoma. We selected cabazitaxel and β-elemene, which have fewer issues with drug resistance, and successfully prepared cabazitaxel liposome, β-elemene liposome and cabazitaxel-β-elemene complex liposome with good flexibility. The encapsulation efficiencies of cabazitaxel and β-elemene in these liposomes were detected by precipitation microfiltration and microfiltration centrifugation methods, respectively. Their encapsulation efficiencies were all above 95%. The release rates were detected by a dialysis method. The release profiles of cabazitaxel and β-elemene in these liposomes conformed to the Weibull equation. The release of cabazitaxel and β-elemene in the complex liposome were almost synchronous. The pharmacodynamics study showed that cabazitaxel flexible liposome and β-elemene flexible liposome were relatively good at overcoming paclitaxel resistance on paclitaxel-resistant lung adenocarcinoma. As the flexible complex liposome, the dosage of cabazitaxel could be reduced to 25% that of the cabazitaxel injection while retaining a similar therapeutic effect. It showed that β-elemene can replace some of the cabazitaxel, allowing the dosage of cabazitaxel to be reduced, thereby reducing the drug toxicity.

## 1. Introduction

Chemotherapy is a common method of tumor treatment. Taxanes, such as paclitaxel and docetaxel, have become the first-line drugs in chemotherapy treatment for conditions such as lung, ovarian and breast cancer [1,2]. However, with the widespread use of paclitaxel, paclitaxel resistance is becoming increasingly prominent and this is one of the main reasons for treatment failure [3,4,5]. Finding a way to overcome the paclitaxel resistance of tumors is imperative. The mechanisms of paclitaxel resistance are complicated, and the occurrence of drug resistance is the result of the complex interaction of multiple factors and pathways [6]. At present, the main ways to overcome the drug resistance of tumor cells are: (1) Combination drug therapy, namely, the combination of two or more drugs for the synergistic treatment of tumors [7,8]; (2) combined use of drug pump inhibitors, such as verapamil and cyclosporine [9,10,11]; (3) using drugs or excipients of reversing drug-resistance, such as elemene [12,13,14], curcumin [15,16,17], and poloxamer [18,19]; (4) using nano-carriers, such as liposomes and nanoparticles [20,21,22]. 

Liposomes are enclosed spherical vesicles composed of lipid bilayers, which were discovered by Bangham and coworkers in 1965 [23,24]. Liposomes as anticancer drug delivery systems have attracted increasing attention, due to their good biocompatibility and biodegradability, and their ability to improve the efficacy and reduce the toxicity of therapeutic drugs. In addition, liposomes can not only encapsulate hydrophilic drugs but also hydrophobic drugs, which could solve the problem of poor water solubility of anti-cancer drugs, such as paclitaxel [25]. Moreover, many studies have confirmed that liposomes can overcome the multidrug resistance of cancers [20,21,26,27]. Flexible liposome is a new type of liposome which is embedded in bilayer membranes of phospholipids such as ethanol, 1,2-propanediol, polysorbate 80 and cholate to reduce the rigidity of phospholipid membranes and increase the fluidity and deformability of membranes [28]. In recent years, combination drug therapy has attracted more and more attention because of its lower toxicity, stronger curative effect and reduced resistance compared to single drug therapy on cancer treatment [29]. Combined treatment of paclitaxel and doxorubicin is a first-line treatment for metastatic breast cancer; this combined treatment has been proved to be more effective than single drug therapy [30]. The U.S. Food and Drug Administration (FDA) approved Vyxeos (also known as CPX-351; Jazz Pharmaceuticals) for the treatment of two types of adult acute myeloid leukemia in 2017 [31]. Vyxeos is a complex liposome injection containing daunorubicin and cytarabine [32].

**Figure 1:**
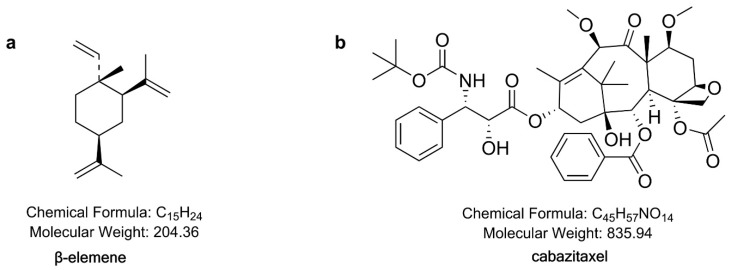
The chemical structure of β-elemene and cabazitaxel.

Cabazitaxel, a semisynthetic taxane, has been approved by the FDA and European Medicines Agency (EMA) for the treatment of castration-resistant prostate cancer with disease progression after docetaxel treatment [33,34]. The antitumor activity spectrum of cabazitaxel in mice bearing human xenografts is broad [35]. However, cabazitaxel is insoluble in water. The Cabazitaxel injection currently on the market (JEVTANA) is solubilized with polysorbate 80, which contains 60 mg cabazitaxel in 1.5 mL polysorbate 80. This injection has serious side effects, such as nausea, vomiting, constipation and renal failure, which limit the clinical application [36]. Recently, Yin et al. [37] developed a polyethylene glycol-modified liposome encapsulating cabazitaxel. The results showed that the cabazitaxel liposome enhanced the inhibitory effect on CT-26 (mouse colon cancer) and T41 (mouse breast cancer) tumors compared to cabazitaxel solution. β-elemene was extracted from the traditional Chinese medicine Curcuma wenyujin Y. H. Chen et C. Ling. Injectable emulsion of elemene has been approved by the China Food and Drug Administration (CFDA) to treat various cancers, including lung, liver, brain, breast, ovary, gastric and prostate cancer [38]. Many studies have shown that β-elemene has a broad foreground in anti-lung cancer applications. It has a certain effect when used alone in lung cancer, but when combined with other anticancer agents, like paclitaxel and cisplatin, β-elemene could enhance the anti-lung cancer effect [39,40]. Moreover, β-elemene could reverse drug resistance when combined with chemotherapeutic drugs [13,14,41,42,43]. Compared to chemotherapeutic drugs, β-elemene has obvious advantages. It can not only inhibit the growth of tumors, but also improve the immune function of the body [39]. To the best of our knowledge, there have been no reports of β-elemene combined with cabazitaxel so far. The chemical structure of β-elemene and cabazitaxel are shown in Figure 1. 

In this work, we developed flexible liposomes to treat paclitaxel-resistant lung adenocarcinoma. According to the theory of molecular compatibility, we chose cabazitaxel and β-elemene as the main drugs, and optimized the drug composition to explore flexible liposome carriers of cabazitaxel, β-elemene and their complex liposome to the treatment of paclitaxel-resistant lung adenocarcinoma, and observed their pharmacodynamics on nude mice with paclitaxel-resistant lung adenocarcinoma.

## 2. Results and Discussion

### 2.1. The Effect of Paclitaxel, Cabazitaxel and β-Elemene on Paclitaxel-Resistant Lung Adenocarcinoma Cells

The effect of paclitaxel, cabazitaxel and β-elemene on lung adenocarcinoma cells (A549) are shown in Table 1. The results showed that paclitaxel was highly resistant to the paclitaxel-resistant lung adenocarcinoma cells (A549/T), and the resistance index was 44.6. Compared with paclitaxel, cabazitaxel showed a significant decrease in the resistance index to the paclitaxel-resistant cells, with a small resistance. Paclitaxel resistance in lung adenocarcinoma cells is currently attributed to repeated drug stimulation and P-glycoprotein pump efflux. During the long-term development of drug resistance, paclitaxel-resistant lung adenocarcinoma cells had changed to adapt to harsh living conditions, such as the emergence of multidrug resistance. Cabazitaxel had a low affinity with P-glycoprotein, so it only demonstrated a small resistance. β-elemene displayed a slight resistance to paclitaxel-resistant lung adenocarcinoma cells. This may be due to the fact that β-elemene had strong permeability. β-elemene also could inhibit the expression of P-glycoprotein [13].

### 2.2. Combined Effect of Overcoming the Resistance of Cabazitaxel and β-Elemene Compositions

The combined effect of overcoming the resistance of cabazitaxel and β-elemene compositions is shown in Table 2. The results showed that the inhibition effects of different ratio compositions of cabazitaxel and β-elemene ranged from 1 to 5.5 times compared with cabazitaxel alone on paclitaxel-resistant lung adenocarcinoma cells (IC_50_ of cabazitaxel alone/IC_50_ of cabazitaxel in compositions). The effects of different ratio compositions of cabazitaxel and β-elemene ranged from 6.4 to 35.3 times compared with that of paclitaxel. The effects of these compositions were significantly higher than that of paclitaxel. When the ratio of composition was greater than 1379.99 μM/174.66 nM (the ratio of β-elemene to cabazitaxel), the effects were increased. When the ratio was 1379.99 μM/43.66 nM the effect was significantly increased to 35.3 times that of paclitaxel, indicating that the composition had a significant effect on overcoming paclitaxel resistance. When the ratios of β-elemene to cabazitaxel were greater, the effect on overcoming paclitaxel resistance was greater. 

### 2.3. Particle Size and Zeta Potential of the Liposomes

According to the results of the combined-effect study and the clinical situation of cabazitaxel injection and elemene injection, the cabazitaxel liposome, β-elemene liposome and the complex liposome with good flexibility were prepared successfully. For passively targeting liposomes, the particle size, zeta potential and permeability were key factors. In these liposomes, d-α-tocopherol polyethylene glycol 1000 succinate (TPGS) was used to enhance the permeability of drugs and reduce the size of liposomes, and the amount of cholesterol was reduced to increase its flexibility further and enable them to cross the barriers of blood vessels and tumors more easily. 

According to the general composition of liposomes, phospholipids are usually 0.5%–3%, cholesterol is 0.1%–1%, TPGS is 0.1%–1%, and ethanol is 0.1%–20% (the dosage of different processes was also different). In the preliminary experiments, we used hydrogenated soybean phospholipids and egg yolk phospholipid. However, their prepared liposomes were not sufficiently transparent compared to that of soybean phospholipid; therefore, soybean phospholipid was chosen as the membrane material of the liposomes. The ratio of cholesterol to soybean phospholipid was reduced to 1:25 in order to enhance the permeability of the complex liposome. TPGS was a good emulsifier, which can enhance the drug permeability and inhibit P-glycoprotein. Therefore, TPGS was chosen as the emulsifying stabilizer. Trehalose was used as an isotonic agent in the prescription. The liposomes prepared by the above prescription had a relatively small particle size, uniform distribution and suitable zeta potential. They were almost transparent. 

The average particle size of these liposomes was caculated by the volume model of Nicomp software v.3.0.6 in particle sizing systems. The average particle size, polydispersity index (PI) value, zeta potential and the mean values of the complex liposome, β-elemene liposome and cabazitaxel liposome are shown in Table 3. According to the features of these liposomes, they may be small unilamellar vesicles. The components between the complex liposome and cabazitaxel liposome were slightly different. The cabazitaxel liposome was freeze-dried, and the size and zeta potential were detected after resolving. Therefore, some differences in their size and zeta potential were observed. The particle size and distribution figures of these liposomes are shown in Figures S1–S9 in the Supplementary Materials.

### 2.4. The Detection of Encapsulation Efficiency of Cabazitaxel and β-Elemene in the Liposomes

The encapsulation efficiency of cabazitaxel in liposomes was determined by the precipitation microfiltration method. The average filter interception recovery of cabazitaxel in the corresponding aqueous solution (n = 6) was 99.72% ± 0.57% and the relative standard deviation (RSD) was 0.58%. Its average total recovery was 100.02% ± 1.05% and the RSD was 1.05%. The average encapsulation efficiency of the cabazitaxel liposome (n = 6) was 95.63% ± 0.67% and the RSD was 0.71%. The average recovery of the cabazitaxel liposome was 102.51% ± 0.91% and the RSD was 0.90%. The average encapsulation efficiency of cabazitaxel in the complex liposome (n = 6) was 96.98% ± 0.85% and the RSD was 0.88%; the average recovery of cabazitaxel in the complex liposome was 98.19% ± 0.27% and the RSD was 0.28%. 

The encapsulation efficiency of β-elemene in liposomes was determined by the microfiltration centrifugation method. The average filter interception recovery of β-elemene in the corresponding aqueous solution (n = 6) was 98.08% ± 0.32% and the RSD was 0.33%. The average encapsulation efficiency of β-elemene liposome (n = 6) was 96.32% ± 0.59% and the RSD was 0.62%. The average recovery of β-elemene liposome was 99.60% ± 1.45% and the RSD was 1.46%. The average encapsulation efficiency of β-elemene in the complex liposome (n = 6) was 99.29% ± 0.42%, with an RSD of 0.43%. The average recovery of β-elemene in the complex liposome was 99.92% ± 0.37% and the RSD was 0.38%. From these results, the method meets the requirements. Their encapsulation efficiencies were all above 95%.

As cabazitaxel did not dissolve easily in water but was easily dissolved in ethanol, the liposome preparation used approximately 1% ethanol (*w*/*w*) as the cosolvent. The free cabazitaxel in the preparation usually dissolved in ethanol or existed in the form of drug particles, and precipitation would occur under the condition of long-term placement. Drugs encapsulated in liposomes were not easy to precipitate. In order to speed up the precipitation of free cabazitaxel, sodium chloride was added to the solution to destroy the physical stability of the free drug and accelerate its precipitation. Then, the precipitation could be removed by a microfiltration membrane. In the process of precipitation formation, the keys were the standing time and the dosage of sodium chloride was the precipitation promoter. Through optimization, the standing time was 4 h and the dosage of sodium chloride was 0.75 g for 5 mL liposome solution. As β-elemene is insoluble in water and is less dense than water, it required more than 50% ethanol solution to completely dissolve. The liposome preparation used approximately 1% ethanol (*w*/*w*) as a cosolvent, which was insufficient to form a stable suspension of β-elemene in aqueous solution. Free β-elemene in the preparation was usually dissolved in trace amounts of ethanol to form tiny oil droplets or floated on the liquid surface. Free β-elemene can be intercepted and removed by 0.45 μm microfiltration centrifugation. The size of liposomes was usually small and they easily passed through the 0.45 μm microfiltration membrane. Therefore, the encapsulation efficiency of β-elemene in liposomes was determined using a 0.45 μm microfiltration centrifugation method. In the pre-experiment, we used the dextran gel column method. However, it was difficult to completely separate free drugs from liposomes. The centrifugation method was also used and the prepared liposomes hardly precipitated, even upon ultra-high-speed centrifugation. Therefore, the precipitation microfiltration method and microfiltration centrifugation method were finally chosen.

### 2.5. The Release Rate Detection of Cabazitaxel and β-Elemene in the Liposomes

An important parameter of liposome is the release rate. It is typically hoped that drugs can achieve synchronized release for the complex liposome so that the drugs can synchronously act on cancer cells. The release rate detection of cabazitaxel and β-elemene in these liposomes are shown in Figures S10–S13 in the Supplementary Materials. The release rate of cabazitaxel in the complex liposome (n = 3) was 16.39% ± 1.13% at 0.5 h, and 85.44% ± 1.18% at 10 h. The release rate of β-elemene in the complex liposome (n = 3) was 5.77% ± 0.63% at 0.5 h, and 99.07% ± 3.3% at 10 h. The release curve of cabazitaxel in the complex liposome conformed to the Weibull equation: F = 100 × [1 − Exp(−t^0.738^/2.713)], r = 0.9985. The release curve of β-elemene in the complex liposome conformed to the Weibull equation: F = 100 × [1 − Exp(−t^1.532^/7.353)], r = 0.9997. The release of cabazitaxel and β-elemene in the complex liposome were almost synchronous. The release rate of cabazitaxel in the cabazitaxel liposome (n = 3) was 23.18% ± 1.49% at 0.5 h, and 86.68% ± 2.69% at 10 h. The release curve of cabazitaxel in the cabazitaxel liposome conformed to the Weibull equation: F = 100 × [1 − Exp(−t^0.624^/2.251)], r = 0.9982. The release rate of β-elemene in the β-elemene liposome (n = 3) was 5.50% ± 0.61% at 0.5 h, and 96.03% ± 1.04% at 10 h. The release curve of β-elemene in the β-elemene liposome conformed to the Weibull equation: F = 100 × [1 − Exp(−t^1.437^/8.61)], r = 0.9997. The release of cabazitaxel in the cabazitaxel liposome and the complex liposome were similar. The release of β-elemene in the β-elemene liposome and the complex liposome were also similar.

### 2.6. The Animal Pharmacodynamics of the Liposomes

**Figure 2:**
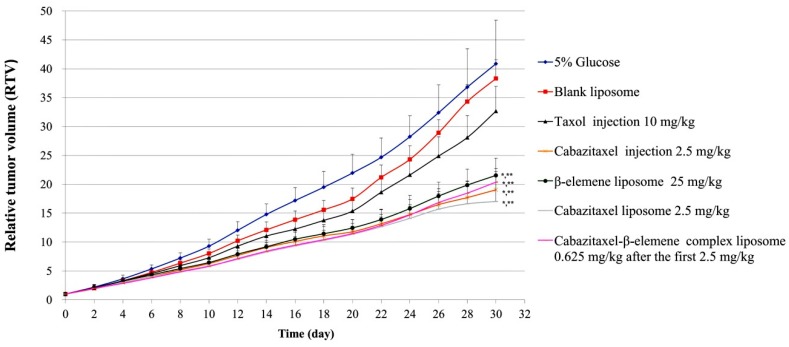
Relative tumor volume profiles of studied liposomes. *Compared with the 5% glucose group, there was a statistically significant difference (*p* < 0.01). Compared with 5% glucose group, the statistics parameter of cabazitaxel injection was t = 7.682, *p* < 0.01; that of the β-elemene liposome group was t = 7.221, *p* < 0.01; that of cabazitaxel liposome was t = 8.012, *p* < 0.01, that of the cabazitaxel-β-elemene complex liposome was t = 8.612, *p* < 0.01. **Compared with the taxol injection group, there was a statistically significant difference (*p* < 0.01). Compared with the taxol injection group, the statistics parameter of cabazitaxel injection was t = 7.373, *p* < 0.01; that of the β-elemene liposome group was t = 6.369, *p* < 0.01; that of the cabazitaxel liposome was t = 7.469, *p* < 0.01; and that of the cabazitaxel-β-elemene complex liposome was t = 8.116, *p* < 0.01. Compared with the cabazitaxel injection group, the statistics parameter of the β-elemene liposome group was t = −1.674, *p* > 0.05; that of the cabazitaxel liposome was t = 1.067, *p* > 0.05; that of the cabazitaxel-β-elemene complex liposome was t = −1.051, *p* > 0.05 (there was no statistically significant difference). Compared with the β-elemene liposome group, the statistics parameter of the cabazitaxel-β-elemene complex liposome group was t = 0.971, *p* > 0.05 (there was no statistically significant difference).

**Figure 3:**
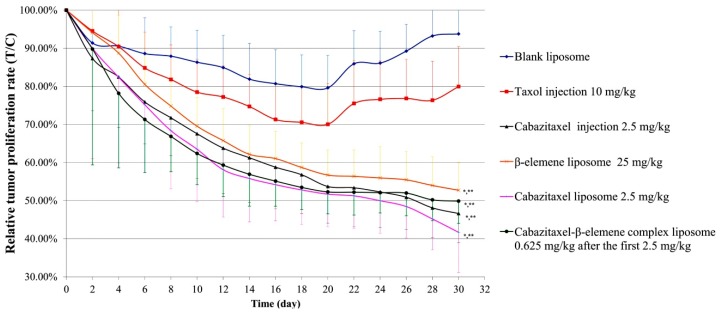
The relative tumor proliferation rate of these liposomes. *Compared with the 5% glucose group, there was a statistically significant difference (*p* < 0.01). **Compared with the taxol injection group, there was a statistically significant difference (*p* < 0.01). Compared with the cabazitaxel injection group, there was no statistically significant difference for the group with cabazitaxel liposome, β-elemene liposome or the complex liposome, respectively. The results of the statistics parameter of the relative tumor proliferation rate were the same as those of the relative tumor volume.

**Figure 4:**
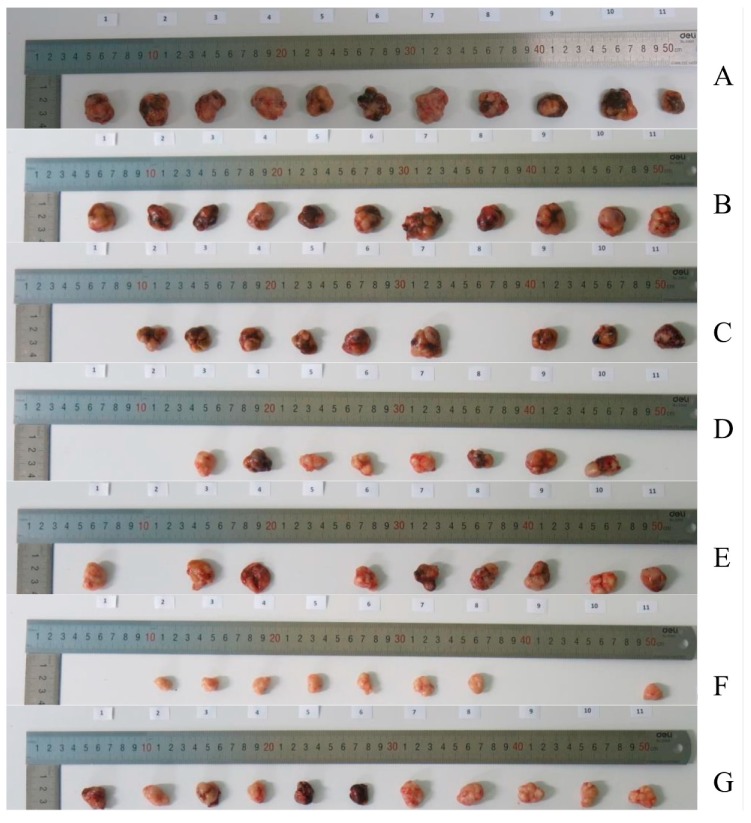
The tumor tissues figure with paclitaxel-resistant lung adenocarcinoma. **A**, 5% glucose; **B**, blank liposome; **C**, taxol injection 10 mg/kg; **D**, cabazitaxel injection 2.5 mg/kg; **E**, β-elemene liposome 25 mg/kg; **F**, cabazitaxel liposome 2.5 mg/kg; **G**, cabazitaxel-β-elemene complex liposome 0.625 mg/kg after the first 2.5 mg/kg.

**Figure 5:**
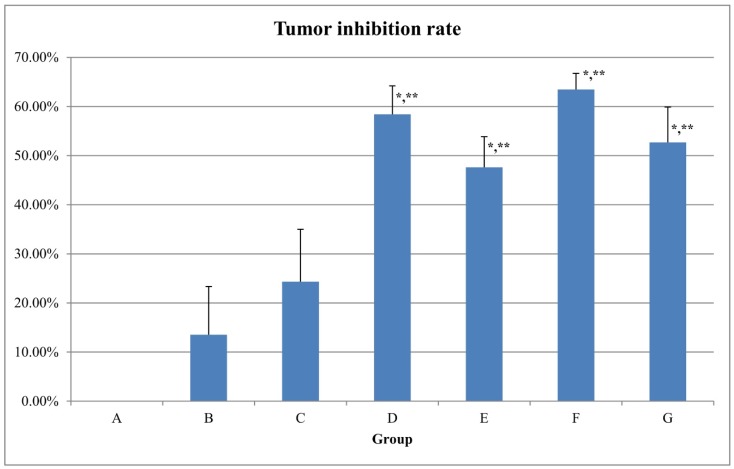
The tumor inhibition rate of the liposomes. A, 5% glucose; B, blank liposome; C, taxol injection 10 mg/kg; D, cabazitaxel injection 2.5 mg/kg; E, β-elemene liposome 25 mg/kg; F, cabazitaxel liposome 2.5 mg/kg; G, cabazitaxel-β-elemene complex liposome 0.625 mg/kg after the first 2.5 mg/kg. *Compared with the 5% glucose group, there was a statistically significant difference (*p* < 0.01). Compared with the 5% glucose group, the statistics parameter of the cabazitaxel injection group was t = −11.870, *p* < 0.01; that of the β-elemene liposome group was t = −10.095, *p* < 0.01; that of the cabazitaxel liposome was t = −15.615, *p* < 0.01; and that of the cabazitaxel-β-elemene complex liposome was t = −11.824, *p* < 0.01. **Compared with the taxol injection group, there was a statistically significant difference (*p* < 0.01). Compared with the taxol injection group, the statistics parameter of the cabazitaxel injection was t = −8.294, *p* < 0.01; that of the β-elemene liposome group was t = −5.648, *p* < 0.01; that of the cabazitaxel liposome was t = −10.461, *p* < 0.01; that of the cabazitaxel-β-elemene complex liposome was t = −7.091, *p* < 0.01. Compared with the cabazitaxel injection group, the statistics parameter of the cabazitaxel-β-elemene complex liposome group was t = 1.842, *p* > 0.05, there was no statistically significant difference. Compared with the β-elemene liposome group, the statistics parameter of the cabazitaxel-β-elemene complex liposome group was t = −1.669, *p* > 0.05, there was no statistically significant difference. The tumor inhibition rates of group B, C, D, E, F, G were 13.53% ± 9.81%, 24.33% ± 10.67%, 58.40% ± 5.81%, 47.62% ± 6.25%, 63.46% ± 3.27%, and 52.71% ± 7.18%, respectively.

Due to the relatively high toxicity of cabazitaxel and the fact that it contained more polysorbate 80 in its marketed preparation, in this study we encapsulated it into liposome. β-elemene liposome and the complex liposome were also prepared, and their therapeutic effects were compared. Considering that the dosage of phospholipids and other excipients in the combination of cabazitaxel liposome and β-elemene liposome was twice as much as that of the complex liposome, both are fat-soluble drugs, and the prescription process was basically the same; therefore, the complex liposome had relative advantages. Thus, this study mainly examined the pharmacodynamics of cabazitaxel liposome, β-elemene liposome and the complex liposome. The relative tumor volume profiles of these liposomes are shown in Figure 2. The relative tumor proliferation rate of these liposomes is shown in Figure 3. The tumor tissues with paclitaxel-resistant lung adenocarcinoma are shown in Figure 4. The tumor inhibition rates of these liposomes are shown in Figure 5. 

From the results, it can be seen that the blank liposome had a slight antitumor effect on nude mice using the human paclitaxel-resistant lung adenocarcinoma model (the drug resistance index was 44.6). Paclitaxel common injection (10 mg/kg) has a certain antitumor effect. The anti-tumor effects of cabazitaxel injection (2.5 mg/kg), β-elemene liposome (25 mg/kg), cabazitaxel liposome (2.5 mg/kg), and the complex liposome (0.625 mg/kg after the first 2.5 mg/kg) were similar. Compared with the group with 5% glucose, these four groups (cabazitaxel injection, β-elemene liposome, cabazitaxel liposome, and the complex liposome) had statistically significant differences. Compared with the taxol injection, these four groups (cabazitaxel injection, β-elemene liposome, cabazitaxel liposome, and the complex liposome) also had statistically significant differences. Compared with the cabazitaxel injection group, there was no statistically significant difference for the cabazitaxel liposome group, β-elemene liposome group or the complex liposome group. Cabazitaxel, which has a low affinity with P-glycoprotein, is a paclitaxel derivative developed to overcome paclitaxel resistance. From the above results, it can be seen that cabazitaxel injection and cabazitaxel liposome all had a relatively good effect on paclitaxel-resistant lung adenocarcinoma. Surprisingly, β-elemene liposome also had a relatively good effect on nude mice with paclitaxel-resistant lung adenocarcinoma. The effect of β-elemene liposome (25 mg/kg) was similar to the effect of cabazitaxel injection (2.5 mg/kg), although β-elemene had a lower inhibitory effect than cabazitaxel in previous cell experiments. This suggests that β-elemene possessed other mechanisms besides directly inhibiting tumor cells. The complex liposome with 0.625 mg/kg cabazitaxel after the first 2.5 mg/kg administered every other day may have a similar effect to that of 2.5 mg/kg cabazitaxel injection every other day or 25 mg/kg β-elemene every day. This implies that, as the flexible complex liposome, the dosage of cabazitaxel could be reduced to 25% that of the cabazitaxel injection while retaining a similar therapeutic effect. It was found that there was no death in mice in the complex liposome group compared with cabazitaxel injection and β-elemene liposome, indicating that the toxicity of the complex liposome was less when the cabazitaxel dosage was reduced. It showed that β-elemene can replace some of the cabazitaxel, reducing the dosage of cabazitaxel, thereby reducing the toxity. This suggests a way to reduce the dosage of cabazitaxel. We speculated that β-elemene with strong permeability and inhibiting p-glycoprotein may be beneficial to overcome the blood vessel barrier, mesenchymal hyperosmotic barrier, and cell membrane barrier of tumors. At the same time, β-elemene had a certain effect in regulating immunity, which may signal related immune molecules to attack tumors and change the microenvironment of tumors, which made cabazitaxel easier to enter into tumor tissue and cells, thus enhancing the efficacy of cabazitaxel. 

Yin et al. [37] developed a PEG-modified liposome encapsulating cabazitaxel (containing egg phospholipid, cholesterol, and PEG2000-DSPE, and chloroform was used in the preparation process). Their result showed that the cabazitaxel liposome enhanced the inhibitory effect on CT-26 (mouse colon cancer) and T41 (mouse breast cancer) tumors compared to the cabazitaxel solution. This result and our results indicate that cabazitaxel has a good efficacy with regard to various tumors, but it is more valuable for drug-resistant tumors. Wang et al. [44] found that β-elemene liposome (containing 6% soybean phospholipid, 1% cholesterol and 1%polyvinylpyrrolidone-k30) had a good inhibitory effect on hepatocellular carcinoma (H22) in nude mice. Wang et al. [45] prepared β-elemene ordinary liposomes, long-circulating liposomes and thermosensitive long-circulating liposomes (containing 5% soybean phospholipid, 1.67% cholesterol, 0.33% PEG2000-DSPE). The results showed that these β-elemene liposomes had a good effect on nude mice with hepatocellular carcinoma (H22). Dong et al. [46] prepared β-elemene-curcumin complex liposomes as atomization inhalation preparation (containing 6.667% phospholipid and 1.333% cholesterol); their results showed that they had a good inhibitory effect on Lewis lung cancer cells in vitro. The above results showed that β-elemene liposome obtained by various prescriptions and preparation techniques has an effect on some tumors. However, the effect of β-elemene liposome, cabazitaxel liposome and cabazitaxel-β-elemene complex liposome on paclitaxel-resistant lung adenocarcinoma was not reported previously. In this study, β-elemene flexible liposome, cabazitaxel flexible liposome and the flexible complex liposome containing TPGS had a relatively good effect on nude mice with paclitaxel-resistant lung adenocarcinoma. It showed that they are worthy of further study. Paclitaxel resistance is very common in clinics. At present, how to overcome paclitaxel resistance is a difficult challenge. In this study, we developed three liposomes to treat paclitaxel-resistant lung adenocarcinoma. From the results, it can be seen that both the cabazitaxel liposome and β-elemene liposome have relatively good anti-tumor effects when used alone, and the dosage of cabazitaxel in the complex liposome can be reduced with a similar therapeutic effect. These results suggest that the above preparations have relatively good clinical potential.

## 3. Materials and Methods 

### 3.1. Instruments

An IX51 biological inverted microscope was purchased from Olympus Co. (Tokyo, Japan). EL-x800 microplate reader was from BioTek Instruments (Winooski, VT, USA). A high-pressure micro-fluidization homogenizer (LM20) from Microfluidics Co. (Westwood, MA, USA) was used. Particle sizing systems (Z3000) was from the Particle Sizing System Co. (Santa Barbara, FL, USA). High-performance liquid chromatography (HPLC, 1260 infinity II) was from Agilent Technologies Inc. (Santa Clara, CA, USA). The chromatographic column (Ultimate PFP, 4.6 × 250 mm, 5 μm) was from Shanghai Welch Materials Co. (Shanghai, China). The centrifugal machine (5424R) was from Eppendorf Co. (Barkhausenweg, Hamburg, Germany).

### 3.2. Materials

A549 (human lung adenocarcinoma cell line) and A549/T (paclitaxel-resistant lung adenocarcinoma cell line) were from Jiangsu Keygen Biotech Co. (Nanjing, Jiangsu, China). RPMI 1640 culture medium was from Jiangsu Keygen Biotech Co. (Nanjing, Jiangsu, China). The fetal bovine serum (FBS, Gibco, 10091-148) was from Thermo Fisher Scientific Co. (Shanghai, China). The thiazolyl blue tetrazolium bromide (MTT) came from Biosharp Co. (Hefei, Anhui, China). Paclitaxel (No. 580555, 97%) was obtained from Sigma Aldrich Co. (Shanghai, China). Cabazitaxel (No. 20170109, 99.9%; No.20180807, 99.6%) was obtained from Wuhan Yuancheng Co-Creation Technology Co., Ltd. (Wuhan, Hubei, China). β-elemene (No. 170704, 94.2%) was from Holistic Integrative Pharmacy Institutes of Hangzhou Normal University (Hangzhou, Zhejiang, China). β-elemene (No. 20180312, 99.1%) was from Hubei Jusheng Technology Co., Ltd. (Wuhan, Hubei, China). β-elemene standard (No. 100268-201402, 99.4%) was obtained from the National Institute for Food and Drug Control (Beijing, China). D-α-tocopherol polyethylene glycol 1000 succinate (TPGS) was from Wuhan GBD Pharm Chemical Co., Ltd. (Wuhan, China). Cholesterol and trehalose were obtained from Shanghai Dingguo Biological Technology Co., Ltd. (Shanghai, China). Soybean phospholipid was from Shanghai Taiwei Pharmaceutical Co., Ltd. (Shanghai, China). Polyvinylidene fluoride (PVDF) microfilter centrifuge tube (0.45 μm, 0.5 mL) was from Merck Millipore Ltd. (Shanghai, China). Syringe filters (0.45 μm, Shanghai Xingya Purification Materials Factory (Shanghai, China). The dialysis bag was from Shanghai Dingguo Biological Technology Co., Ltd. (Shanghai, China). BALB/c nude mice were from Shanghai SLAC Laboratory Animal Co. Ltd. (Shanghai, China), experimental animal production license: SCXK (Shanghai) 2017-0005, qualified number: 2015000565714, experimental animals use license: SLXK (Shanghai) 2017-0015, 4–5 weeks, female.

### 3.3. Combined Effect of Overcoming Paclitaxel Resistance of Cabazitaxel and β-Elemene Compositions

The cryopreserved A549 and A549/T were recovered and transferred to the cell culture flask containing the culture medium. The cells were distributed uniformly by gently shaking. The culture medium was RPMI 1640 with 10% fetal bovine serum. The cell culture bottle was cultured in a CO_2_ incubator. After cell passage, the cells were observed to have no abnormalities and prepared to be inoculated. A549 and A549/T were digested and counted. The cell density was about 6 × 10^5^/mL. The above cells were added to the medium containing serum, and 100 μL of cell suspensions were added into a 96-well cell culture plate to obtain 3000 cells in each well. Then, it was incubated for 24 h at 37 °C in a 5% CO_2_ incubator. Drug solutions of different ratios of cabazitaxel/β-elemene (26.32 nM/714.46 μM, 13.16 nM/714.46 μM, 6.58 nM/714.46 μM, 26.32 nM/357.23 μM, 26.32 nM/178.62 μM for A549 cell; 174.66 nM/1379.99 μM, 87.33 nM/1379.99 μM, 43.66 nM/1379.99 μM, 174.66 nM/689.99 μM, 174.66 nM/345.00 μM for A549/T cell) were prepared by using dimethyl sulfoxide (DMSO) as the solvent. The drug solutions were diluted to the desired content with culture medium. A quantity of 100 μL of the diluted drug medium was added into the well. A negative control group was also established. The 96-well cell culture plate was incubated for 72 h at 37 °C in 5% CO_2_. Ninety-six-well cell culture plates were stained with MTT and their optical density (OD) values were detected according to the following steps: 20 μL of MTT solution (5 mg/mL) was added to each well and incubated for 4 h in the incubator. The supernatant was discarded. A quantity of 150 μL DMSO was added to each well and gently mixed for 10 min in the shaking table. The OD value of each well was detected by the plate reader, λ was 490 nm. Then the inhibition rate was calculated according this formula:

Inhibition rate (%) = (OD value of negative control group − OD value of experimental group)/OD value of negative control group × 100%(1)

The IC_50_ (half maximal inhibitory concentration) values of the drugs were calculated by SPSS 18.0 software (IBM SPSS Statistics, Chicago, IL, USA).

### 3.4. The Preparation of Cabazitaxel Liposome, β-Elemene Liposome and Their Complex Liposome

According to the results of the combined-effect study, when the clinical situation of cabazitaxel injection and elemene injection were considered at the same time, the formulation and process of cabazitaxel-β-elemene complex liposome were as follows: 160 mg cabazitaxel was weighed and dissolved by adding 9.5 g ethanol. Then, 4 g β-elemene and the appropriate excipients such as 0.8 g cholesterol, 20 g soybean phospholipid and 4 g TPGS were added and dissolved by heating at 80 °C as the organic phase. A total of 80 g trehalose was dissolved into 682 g water and preserved at 60 °C as the aqueous phase. The organic phase was added into the aqueous phase. Then, the suspension was sheared at 15,000 r/min for 1 h, and high-pressure homogeneity was performed three times at 15,000 psi. Then, the complex liposome was prepared. The preparation method and prescription of β-elemene liposome were the same except that no cabazitaxel was used. The formulation and process of cabazitaxel liposome were as follows: 400 mg cabazitaxel, 0.48 g cholesterol, 12 g soy phospholipid, and 4 g TPGS, were dissolved into 100 mL ethanol, then it was rotated and vacuum-pumped to remove ethanol at 60 °C. A total of 160 g trehalose was dissolved in 623 g water to dissolve the above materials. Then, it was sheared at 15,000 r/min for 1 h, and high-pressure homogeneity was performed three times at 15,000 psi. Finally, the cabazitaxel liposome was freeze-dried. As the cabazitaxel liposome had no β-elemene, the dosage of cabazitaxel may be increased and the dosage of phospholipid may be reduced. The complex liposome and β-elemene liposome were not freeze-dried because of the volatility of β-elemene. The particle size was detected by Nicomp software v.3.0.6 of particle sizing systems. The common market-dosage of cabazitaxel was 25 mg/m^2^ according to the human body surface area. The common dosage of elemene injection in the market was 400–600 mg each day (about 80–100 mL each day, an emulsion injection containing soybean phospholipid). Considering the clinical use, we prepared the complex liposomes with 0.2 mg/mL of cabazitaxel and 5 mg/mL of β-elemene.

### 3.5. The Content Detection of Cabazitaxel and β-Elemene in the Liposomes

The cabazitaxel and β-elemene contents were detected by HPLC according to the following conditions: The detection wavelength was 230/210 nm and the flow rate was 0.9 mL/min. For the preparation of phosphoric acid water, 1000 mL water was adjusted to a pH of 4.0 by adding 1% phosphoric acid aqueous solution. The mobile phase A was methanol/acetonitrile/phosphoric acid water (25:30:45), the mobile phase B was methanol/acetonitrile/phosphoric acid water (25:55:20), and the mobile phase C was methanol/acetonitrile/phosphoric acid water (5:90:5). The gradient elution conditions of HPLC were as follows: at 0 min, the mobile phase A/B was 80/20; at 45 min, the mobile phase A/B was 20/80; at 50 min, C was 100. The detection time was 60 min. The injection volume was 20 μL and the column temperature was 30 °C. The detection of cabazitaxel in the liposomes was as follows: 1 mL of the liposome solution was placed in a 25 mL volumetric flask, and methanol was added to the scale of the volumetric flask, which then was subjected to sonication for 30 min and shaken well after cooling. Then, cabazitaxel in the liposome was detected by liquid chromatography conditions at 230 nm, as described above. The detection of β-elemene in the liposomes was as follows: 1 mL of the liposome was placed in a 25 mL volumetric flask, and methanol was added to the scale of the volumetric flask, which then was subjected to sonication for 30 min and shaken well after cooling. Then, 1 mL of this solution was taken out and diluted to 10 mL with methanol and shaken well. Then, β-elemene in the liposome was detected by liquid chromatography conditions at 210 nm, as described above.

It was found that the excipients of the liposome did not interfere with the detection of cabazitaxel and β-elemene. The retention time was about 14 min for cabazitaxel. The theoretical plate number of cabazitaxel was more than 4000, and the chromatographic resolution of cabazitaxel was more than 1.5. The cabazitaxel reference solution was linear in the range of 0.30–100.00 μg/mL. The regression equation of cabazitaxel was y = 25.52798x, r = 0.99999. The retention time was about 31 min for β-elemene. The theoretical plate number of β-elemene was more than 4000, and the chromatographic resolution of β-elemene was more than 1.5. The β-elemene reference solution was linear in the range of 0.80–120.00 μg/mL. The regression equation of β-elemene was y = 18.03583x, r = 0.99999.

### 3.6. The Detection of Encapsulation Efficiency of Cabazitaxel and β-Elemene in the Liposomes

The detection method of encapsulation efficiency of cabazitaxel in liposome was as follows: 5 mL liposome solution was placed into a centrifugal tube and 0.75 g sodium chloride was added and dissolved by vortex oscillating for 3 min, then reserved at 25 °C for 4 h. Then, it was transferred to a 10 mL syringe and was filtered with a 0.45 μm hydrophilic syringe filter. The filtrate was added to 50 mL of methanol and dissolved by sonication. Its content was determined by HPLC, which was given as A. The 10 mL syringe was washed with 20 mL methanol and the syringe filter was immersed in the methanol washing fluid and sonicated for 30 min, cooled and added to 25 mL of methanol. Its content was determined by HPLC, which was given as B. The cabazitaxel content in the liposome was given as Z. The encapsulation efficiency of cabazitaxel in liposome was equal to A/(A + B) × 100%, and the total recovery was equal to (A + B)/Z × 100%. The corresponding aqueous solution of cabazitaxel was prepared according to the liposome prescription. The corresponding aqueous solution of cabazitaxel was taken to be 5 mL, and then processed according to the above method. The content of filtrate was given as C. The cabazitaxel content in the syringe and filter was given as D. The filter interception recovery of cabazitaxel in the corresponding aqueous solution was equal to D/(C + D) × 100%.

The detection method of the encapsulation efficiency of β-elemene in liposome: 0.2 mL of the liposome was put into a centrifuge tube with a 0.45 μm PVDF microfiltration membrane and was centrifuged for 10 min at 20 °C and 12,000× *g*. The lower liquid in the centrifuge tube was taken out and 50 mL of methanol was added; its content was given as A. The upper micro filter tube in the centrifuge tube was taken out and added to 20 mL of methanol and sonicated for 30 min, and then added to 25 mL of methanol; its content was given as B. The β-elemene content in the liposome was given as Z. The encapsulation efficiency of β-elemene was equal to A/(A + B) × 100%. The total recovery was equal to (A + B)/Z × 100%. The corresponding aqueous solution of β-elemene was prepared according to the liposome prescription. The corresponding aqueous solution of β-elemene was taken to be 0.2 mL and was then processed according to the above method. The content of the lower layer of the tube was given as C. The content of the upper layer of tube was given as D. The filter interception recovery of β-elemene in the corresponding aqueous solution was equal to D/(C + D) × 100%.

### 3.7. The Release Rate Detection of Cabazitaxel and β-Elemene in the Liposomes

As β-elemene was insoluble in water, it needed more than 50% ethanol solution to completely dissolve. Both cabazitaxel and β-elemene were easily soluble in 75% ethanol; therefore, 75% ethanol as a dialysis medium may achieve a leaky groove condition. The detection method of the release rate was as follows: 10 mL of liposome solution was put into a dialysis bag (MW 300000) and placed in a dialysate (75% ethanol 100 mL), and was stirred at 300 r/min and 37 °C. A total of 2 mL of dialysate was taken at 0, 0.5, 1, 2, 4, 6, 8, and 10 h (2 mL of new dialysate was added after each sampling). The content of cabazitaxel and β-elemene in the taken dialysate were detected. The cumulative release rate of cabazitaxel and β-elemene in the liposomes was calculated. The release of each liposome was tested three times.

### 3.8. The Animal Pharmacodynamics of the Liposomes

The animal experiments were approved by the Scientific Research Ethics Committee of Hangzhou Normal University (number: 2017-030), in accordance with the guiding opinions on the treatment of laboratory animals in China (2006-398).

Paclitaxel-resistant A549/T cells (the drug resistance index was 44.6), with a cell concentration of about 1 × 10^7^/mL, were injected subcutaneously into the right axilla of each nude mouse. The drug was administered when the tumor volume was about 100 mm^3^. A total of 77 nude mice were randomly divided into seven groups. In total, 11 nude mice were in each group. The drug administration method was slow intravenous administration via a caudal vein. The taxol injection group used 10 mg/kg, administered every other day. The cabazitaxel injection group used 2.5 mg/kg, administration every other day. The β-elemene liposome group used 25 mg/kg, administered every day. The cabazitaxel liposome group used 2.5 mg/kg, administered every other day. The cabazitaxel-β-elemene complex liposome group used 2.5 mg/kg for the first dose, and 0.625 mg/kg for the later dose, administered every other day. The solvent group used a 5% glucose injection, administered every other day. The blank liposome group used blank liposome, administered every other day. The tumor volume was measured once every two days and the relative tumor proliferation rate was calculated. After 30 days, the tumor tissues were taken and weighed to calculate the tumor inhibition rate. The tumor volume was equal to 0.5 × a × b^2^; a is the long diameter of tumor and b is the short diameter of tumor. The relative tumor volume (RTV) was equal to Vt/V0, where V0 is the tumor volume at zero days, and Vt is the tumor volume measured at each time. The formula of relative tumor proliferation rate (T/C) was as follows: (the relative tumor volume of the treatment group/the relative tumor volume of the control group) × 100%. The formula of the tumor inhibition rate was as follows: (1 − the tumor weight of the treatment group/the tumor weight of the control group) × 100%. The statistical analysis of the results was performed using a *t*-test on the SPSS 18.0 software.

## 4. Conclusions

The cabazitaxel liposome, β-elemene liposome and the complex liposome were prepared successfully. The encapsulation efficiencies of drugs in the liposomes were detected using a new precipitation microfiltration or microfiltration centrifugation method. Their encapsulation efficiencies were all above 95%. The release rates were detected using a dialysis method. The release profiles of cabazitaxel and β-elemene in these liposomes conformed to the Weibull equation. The release of cabazitaxel and β-elemene in the complex liposome was almost synchronous. A pharmacodynamics study showed that cabazitaxel liposome and β-elemene liposome had relatively good effects on overcoming paclitaxel resistance in paclitaxel-resistant lung adenocarcinoma. As the flexible complex liposome, the dosage of cabazitaxel could be reduced to 25% that of the cabazitaxel injection while retaining a similar therapeutic effect. The results showed that β-elemene can replace some of the cabazitaxel and reduced the dosage of cabazitaxel, thereby reducing the drug toxicity.

## Figures and Tables

**Table 1:** The resistance index of paclitaxel, cabazitaxel and β-elemene.

Drug		A549	A549/T
paclitaxel	IC_50_ (nM)	25.131	1121.433
paclitaxel	resistance index	/	44.6
cabazitaxel	IC_50_ (nM)	25.771	174.501
cabazitaxel	resistance index	/	6.8
β-elemene	IC_50_ (μM)	757.343	1384.027
β-elemene	resistance index	/	1.8

**Table 2:** Combined effect of overcoming resistance of cabazitaxel and β-elemene compositions.

Cabazitaxel(nM): β-Elemene(μM) (for A549)	Cabazitaxel(nM): β-Elemene(μM) (for A549/T)	Cabazitaxel and β-Elemene Composition	A549	A549/T
/	/	the multiple of increased effect of cabazitaxel to paclitaxel (it was IC_50_ of paclitaxel/IC_50_ of cabazitaxel)	1.0	6.4
26.32/714.46	174.66/1379.99	IC_50_ (nM) of cabazitaxel in composition	10.855	61.235
		IC_50_ (μM) of β-elemene in composition	294.682	483.827
		the multiple of increased effect of cabazitaxel to paclitaxel	2.3	18.3
13.16/714.46	87.33/1379.99	IC_50_ (nM) of cabazitaxel in composition	6.661	42.342
		IC_50_ (μM) of β-elemene in composition	361.647	669.104
		the multiple of increased effect of cabazitaxel to paclitaxel	3.8	26.5
6.58/714.46	43.66/1379.99	IC_50_ (nM) of cabazitaxel in composition	5.167	31.745
		IC_50_ (μM) of β-elemene in composition	561.046	1003.306
		the multiple of increased effect of cabazitaxel to paclitaxel	4.9	35.3
26.32/357.23	174.66/689.99	IC_50_ (nM) of cabazitaxel in composition	20.073	120.685
		IC_50_ (μM) of β-elemene in composition	272.469	476.775
		the multiple of increased effect of cabazitaxel to paclitaxel	1.3	**9.3**
26.32/178.62	174.66/345.00	IC_50_ (nM) of cabazitaxel in composition	21.338	175.360
		IC_50_ (μM) of β-elemene in composition	144.816	346.388
		the multiple of increased effect of cabazitaxel to paclitaxel	1.2	6.4

**Table 3:** The particle size and zeta potential of the liposomes. PI = Poydispersity index.

Drug	No	Diameter (nm)	St.Dev (nm)	PI	Zeta Potential	Mean Diameter (n = 3)	Mean PI (n = 3)	Mean Zeta Potential (n = 3)
Complex liposome	1	65.04	27.84	0.18	−28.06	62.29 ± 2.42	0.19 ± 0.01	−28.33 ± 0.96
	2	61.35	27.55	0.20	−29.39			
	3	60.48	27.34	0.20	−27.53			
β-elemene liposome	1	67.22	30.85	0.21	−33.81	66.29 ± 1.35	0.21 ± 0.01	−32.91 ± 1.48
	2	66.91	31.32	0.22	−33.73			
	3	64.75	29.72	0.21	−31.20			
Cabazitaxel liposome	1	135.87	64.54	0.23	−42.25	132.21 ± 5.77	0.23 ± 0.03	−41.34 ± 1.06
	2	125.56	62.66	0.25	−40.18			
	3	135.21	60.71	0.20	−41.58			

## Supplementary Materials

The following are available online, Figure S1–S3, the particle size and distribution of the complex liposome. Figure S4–S6, the particle size and distribution of the β-elemene liposome. Figure S7–S9, the particle size and distribution of the cabazitaxel liposome. Figure S10, the release rate and fitting curve of cabazitaxel in the complex liposome. Figure S11, the release rate and fitting curve of β-elemene in the complex liposome. Figure S12, the release rate and fitting curve of cabazitaxel in the cabazitaxel liposome. Figure S13, the release rate and fitting curve of β-elemene in the β-elemene liposome.
