# Supplementary material for: The Preparation, Determination of a Flexible Complex Liposome Co-Loaded with Cabazitaxel and β-Elemene, and Animal Pharmacodynamics on Paclitaxel-Resistant Lung Adenocarcinoma
Source: Molecules. 2019 Apr 30;24(9):1697. doi: 10.3390/molecules24091697 (PMC6539285; doi:10.3390/molecules24091697)
Supplement: Supplementary file 1 [file molecules-24-01697-s001.pdf]

Supporting Information

The Preparation, Determination of a Flexible Complex Liposome Co-Loaded with Cabazitaxel and β-Elemene, and Animal Pharmacodynamics on Paclitaxel-Resistant Lung Adenocarcinoma

Yi-Ying Zeng [1,2,3,4], Yi-Jun Zeng [2,3,4], Na-Na Zhang [2,3,4], Chen-Xi Li [2,3,4], Tian Xie [1,2,3,4,*] and Zhao-Wu Zeng [2,3,4,*]

[1] Institute of Chinese Materia Medica, Shanghai University of Traditional Chinese Medicine, 1200 Cailun Road, Shanghai 201203, China; zengyiying2008@163.com
[2] Holistic Integrative Pharmacy Institutes, Hangzhou Normal University, 1378 Wenyi Road, Hangzhou 311121, China; zy0508j@163.com (Y.-J.Z.); nanazhang2019@126.com (N.-N.Z.); chenxichenhaochen@163.com (C.-X.L.)
[3] Key Laboratory of Elemene Class Anti-cancer Chinese Medicine of Zhejiang Province, Hangzhou 311121, China
[4] Engineering Laboratory of Development and Application of Traditional Chinese Medicine from Zhejiang Province, Hangzhou 311121, China
* Correspondence: xbs@hznu.edu.cn (T.X.); artgreenking@126.com (Z.-W.Z.); Tel.: +86 571 28860237 (T.X.); +86 571 28865458 (Z.-W.Z.)

Contents

Figure S1–S3, the particle size and distribution of the complex liposome.

Figure S4–S6, the particle size and distribution of the β-elemene liposome.

FigureS7–S9, the particle size and distribution of the cabazitaxel liposome.

Figure S10, the release rate and fitting curve of cabazitaxel in the complex liposome

Figure S11, the release rate and fitting curve of β-elemene in the complex liposome

Figure S12, the release rate and fitting curve of cabazitaxel in the cabazitaxel liposome

Figure S13, the release rate and fitting curve of β-elemene in the β-elemene liposome

Particle Size Analysis

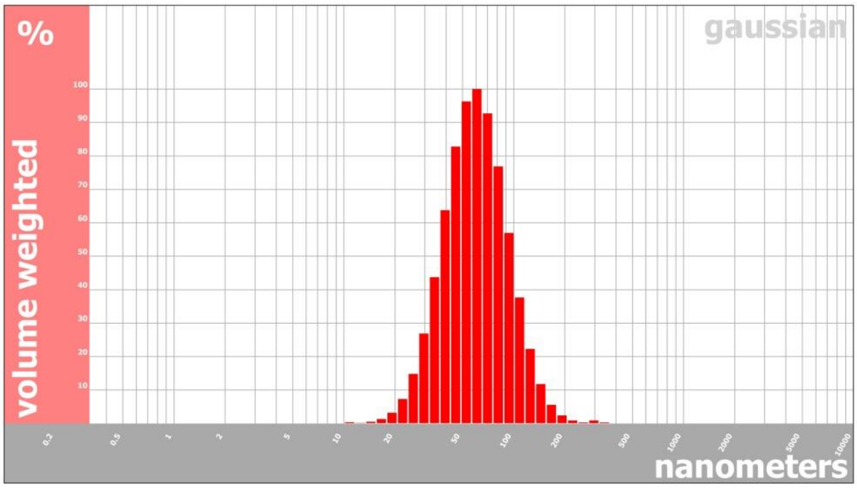

Particle Sizing System NICOMP **Serial Number** 1808303

cabazitaxel-beta-elemene liposome - Lot: 2018111701

Custom Recipe

**Sample Date**	2019/4/18	**Measurement Date**	2019/4/18 13:14 UTC+08:00
**Refractive Index**	1.333	**Channel Width**	13 µsec (auto)
**Viscosity**	0.933 cP	**Temperature**	23 °C
**Particle Type**	Solid	**Intensity Set Point**	300 KHz
**Cell Type**	Square	**First Channel Used**	2

Analysis Results - Gaussian Distribution

	Intensity	Volume	Number
**Mean Diameter**	**108.08**	**65.04**	**36.41**
St.Dev.	46.26	27.84	15.58
CV %	42.80%	42.80%	42.80%
PI	0.18	0.18	0.18

**Cumulative Results**	**Intensity**	**Volume**	**Number**
25% of distribution <	73.79	44.42	25.14
50% of distribution <	98.53	59.31	33.40
75% of distribution <	131.57	79.18	44.49
80% of distribution <	141.34	85.06	47.78
90% of distribution <	170.66	102.71	57.66
99% of distribution <	266.96	160.67	90.10

**Run Time**	0:05:03
**Chi²**	15.755
**Baseline Adj.**	0.000
**Decay**	2.487
**Fit Error**	5.991
**Residual**	5.439
**Minimun Diameter**	10 nm
**Plot Size**	45
**Smoothing**	3
**Plot Range**	100

Printed:
2019/4/18 13:15 UTC+08:00 - zyy

Measured:
2019/4/18 13:14 UTC+08:00 - zyy
Approved:
2019/4/18 13:14 UTC+08:00 - zzw

Figure S1. The particle size and distribution of the complex liposome (1)

Particle Size Analysis

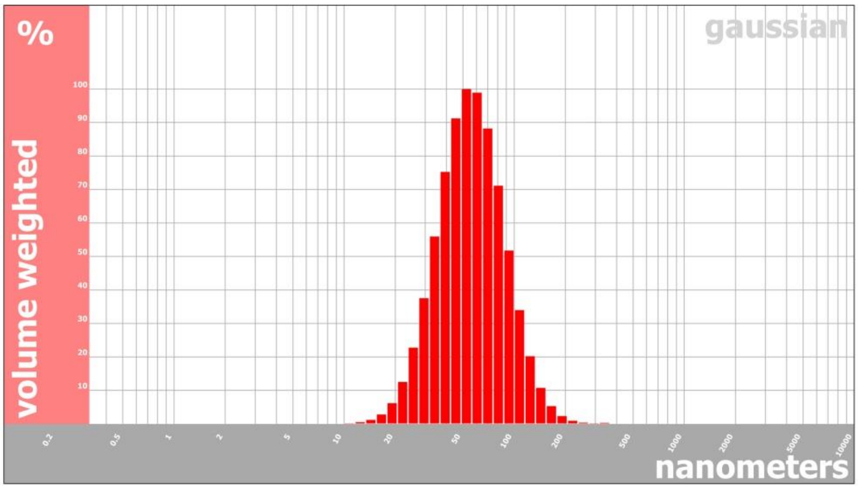

Particle Sizing System NICOMP **Serial Number** 1808303

cabazitaxel-beta-elemene liposome - Lot: 2018111701

Custom Recipe

**Sample Date**	2019/4/18	**Measurement Date**	2019/4/18 13:23 UTC+08:00
**Refractive Index**	1.333	**Channel Width**	12 μsec (auto)
**Viscosity**	0.933 cP	**Temperature**	23 °C
**Particle Type**	Solid	**Intensity Set Point**	300 KHz
**Cell Type**	Square	**First Channel Used**	2

Analysis Results - Gaussian Distribution

	Intensity	Volume	Number
**Mean Diameter**	**107.62**	**61.35**	**32.55**
St.Dev.	48.32	27.55	14.61
CV %	44.90%	44.90%	44.90%
PI	0.20	0.20	0.20

Cumulative Results	Intensity	Volume	Number
25% of distribution <	71.77	40.94	22.34
50% of distribution <	97.21	55.43	29.86
75% of distribution <	131.66	75.07	40.21
80% of distribution <	141.93	80.92	43.31
90% of distribution <	172.98	98.62	52.69
99% of distribution <	276.60	157.70	84.05

**Run Time**	0:05:03
**Chi²**	23.420
**Baseline Adj.**	0.000
**Decay**	2.307
**Fit Error**	7.519
**Residual**	0.000
**Minimun Diameter**	10 nm
**Plot Size**	45
**Smoothing**	3
**Plot Range**	100

Printed:
2019/4/18 13:24 UTC+08:00 - zyy

Measured:
2019/4/18 13:23 UTC+08:00 - zyy
Approved:
2019/4/18 13:23 UTC+08:00 - zzw

Figure S2. The particle size and distribution of the complex liposome (2)

Particle Size Analysis

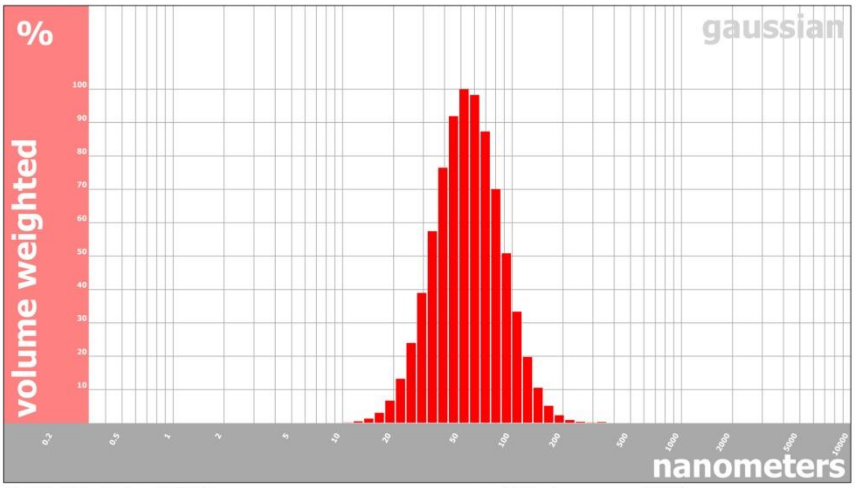

Particle Sizing System NICOMP **Serial Number** 1808303

cabazitaxel-beta-elemene liposome - Lot: 2018111701

Custom Recipe

**Sample Date**	2019/4/18	**Measurement Date**	2019/4/18 13:31 UTC+08:00
**Refractive Index**	1.333	**Channel Width**	12 µsec (auto)
**Viscosity**	0.933 cP	**Temperature**	23 °C
**Particle Type**	Solid	**Intensity Set Point**	300 KHz
**Cell Type**	Square	**First Channel Used**	2

Analysis Results - Gaussian Distribution

	Intensity	Volume	Number
**Mean Diameter**	**106.93**	**60.48**	**31.89**
St.Dev.	48.33	27.34	14.41
CV %	45.20%	45.20%	45.20%
PI	0.20	0.20	0.20

**Cumulative Results**	**Intensity**	**Volume**	**Number**
25% of distribution <	71.10	40.24	21.89
50% of distribution <	96.47	54.58	29.27
75% of distribution <	130.89	74.05	39.47
80% of distribution <	141.17	79.87	42.52
90% of distribution <	172.26	97.45	51.78
99% of distribution <	276.23	156.26	82.81

**Run Time**	0:05:02
**Chi²**	15.661
**Baseline Adj.**	0.000
**Decay**	2.323
**Fit Error**	5.652
**Residual**	18.254
**Minimun Diameter**	10 nm
**Plot Size**	45
**Smoothing**	3
**Plot Range**	100

Printed:
2019/4/18 13:32 UTC+08:00 - zyy

Measured:
2019/4/18 13:31 UTC+08:00 - zyy
Approved:
2019/4/18 13:31 UTC+08:00 - zzw

Figure S3. The particle size and distribution of the complex liposome (3)

Particle Size Analysis

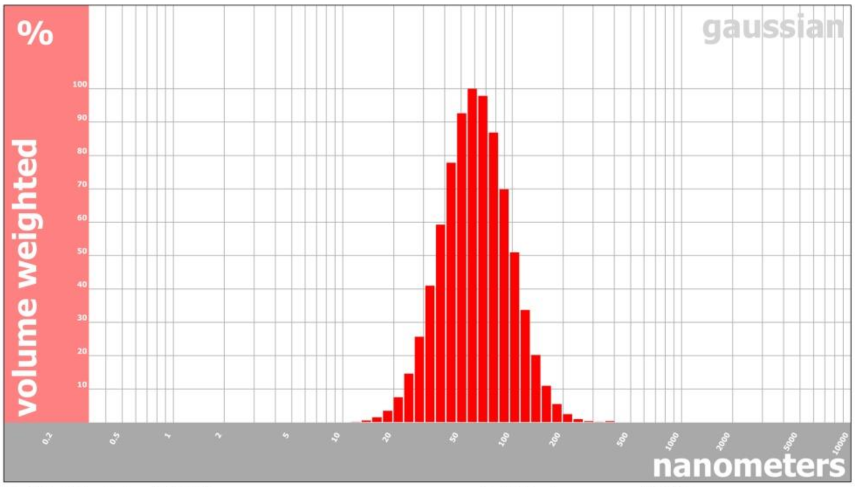

Particle Sizing System NICOMP | **Serial Number** 1808303

beta-elemene liposome - Lot: 2018110801

Custom Recipe

**Sample Date**	2019/4/18	**Measurement Date**	2019/4/18 12:31 UTC+08:00
**Refractive Index**	1.333	**Channel Width**	13 µsec (auto)
**Viscosity**	0.933 cP	**Temperature**	23 °C
**Particle Type**	Solid	**Intensity Set Point**	300 KHz
**Cell Type**	Square	**First Channel Used**	2

Analysis Results - Gaussian Distribution

	Intensity	Volume	Number
**Mean Diameter**	**119.31**	**67.22**	**34.40**
St.Dev.	54.76	30.85	15.79
CV %	45.90%	45.90%	45.90%
PI	0.21	0.21	0.21

Cumulative Results	Intensity	Volume	Number
25% of distribution <	78.69	44.37	23.64
50% of distribution <	107.29	60.47	31.64
75% of distribution <	146.27	82.43	42.78
80% of distribution <	157.95	89.01	46.14
90% of distribution <	193.33	108.94	56.33
99% of distribution <	312.34	175.99	90.67

**Run Time**	0:05:02
**Chi²**	84.693
**Baseline Adj.**	0.000
**Decay**	2.253
**Fit Error**	17.458
**Residual**	11.261
**Minimun Diameter**	10 nm
**Plot Size**	45
**Smoothing**	3
**Plot Range**	100

Printed:
2019/4/18 12:37 UTC+08:00 - zyy

Measured:
2019/4/18 12:31 UTC+08:00 - zyy
Approved:
2019/4/18 12:31 UTC+08:00 - zzw

Figure S4. The particle size and distribution of the β-elemene liposome (1)

Particle Size Analysis

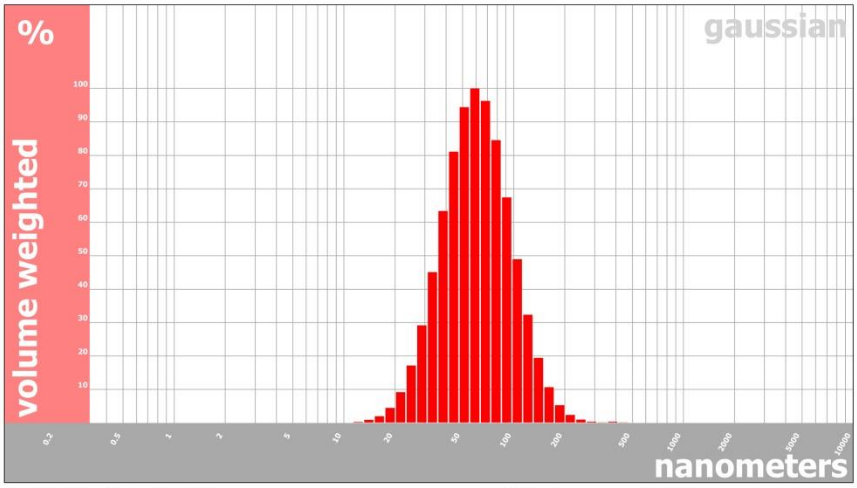

Particle Sizing System NICOMP | **Serial Number** 1808303

beta-elemene liposome - Lot: 2018110801

Custom Recipe

**Sample Date**	2019/4/18	**Measurement Date**	2019/4/18 12:46 UTC+08:00
**Refractive Index**	1.333	**Channel Width**	13 µsec (auto)
**Viscosity**	0.933 cP	**Temperature**	23 °C
**Particle Type**	Solid	**Intensity Set Point**	300 KHz
**Cell Type**	Square	**First Channel Used**	2

Analysis Results - Gaussian Distribution

	**Intensity**	**Volume**	**Number**
**Mean Diameter**	**121.23**	**66.91**	**33.46**
St.Dev.	56.73	31.32	15.66
CV %	46.80%	46.80%	46.80%
PI	0.22	0.22	0.22

**Cumulative Results**	**Intensity**	**Volume**	**Number**
25% of distribution <	79.17	43.75	23.09
50% of distribution <	108.58	59.97	30.89
75% of distribution <	148.90	82.22	41.90
80% of distribution <	161.01	88.91	45.23
90% of distribution <	197.83	109.23	55.38
99% of distribution <	322.50	178.05	89.84

**Run Time**	0:05:02
**Chi²**	90.537
**Baseline Adj.**	0.000
**Decay**	2.218
**Fit Error**	15.020
**Residual**	13.089
**Minimun Diameter**	10 nm
**Plot Size**	45
**Smoothing**	3
**Plot Range**	100

Printed:
2019/4/18 12:48 UTC+08:00 - zyy

Measured:
2019/4/18 12:46 UTC+08:00 - zyy
Approved:
2019/4/18 12:46 UTC+08:00 - zzw

Figure S5. The particle size and distribution of the β-elemene liposome (2)

Particle Size Analysis

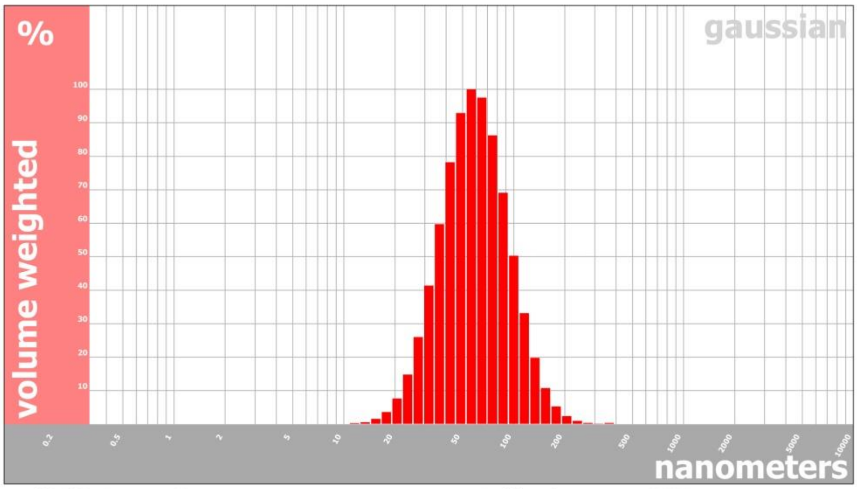

Particle Sizing System NICOMP | **Serial Number** 1808303

beta-elemene liposome - Lot: 2018110801

Custom Recipe

**Sample Date**	2019/4/18	**Measurement Date**	2019/4/18 13:05 UTC+08:00
**Refractive Index**	1.333	**Channel Width**	13 μsec (auto)
**Viscosity**	0.933 cP	**Temperature**	23 °C
**Particle Type**	Solid	**Intensity Set Point**	300 KHz
**Cell Type**	Square	**First Channel Used**	2

Analysis Results - Gaussian Distribution

	Intensity	Volume	Number
**Mean Diameter**	**115.45**	**64.75**	**33.28**
St.Dev.	52.99	29.72	15.28
CV %	45.90%	45.90%	45.90%
PI	0.21	0.21	0.21

**Cumulative Results**	Intensity	Volume	Number
25% of distribution <	76.16	42.75	22.88
50% of distribution <	103.83	58.25	30.61
75% of distribution <	141.54	79.40	41.39
80% of distribution <	152.84	85.74	44.64
90% of distribution <	187.06	104.93	54.49
99% of distribution <	302.15	169.48	87.71

**Run Time**	0:05:02
**Chi²**	77.978
**Baseline Adj.**	0.000
**Decay**	2.330
**Fit Error**	17.560
**Residual**	10.814
**Minimun Diameter**	10 nm
**Plot Size**	45
**Smoothing**	3
**Plot Range**	100

Printed:
2019/4/18 13:06 UTC+08:00 - zyy

Measured:
2019/4/18 13:05 UTC+08:00 - zyy
Approved:
2019/4/18 13:05 UTC+08:00 - zzw

Figure S6. The particle size and distribution of the β-elemene liposome (3)

Particle Size Analysis

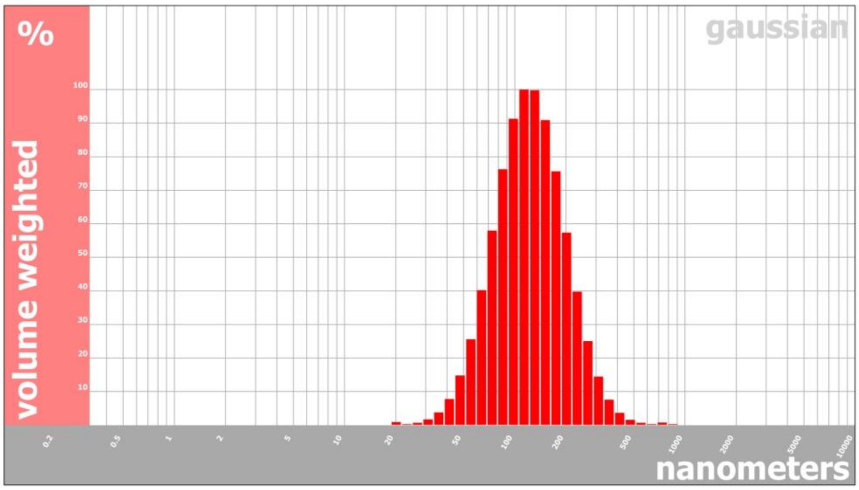

Particle Sizing System NICOMP | **Serial Number** 1808303

cabazitaxel liposome - Lot: 2018111401

Custom Recipe

**Sample Date**	2019/4/18	**Measurement Date**	2019/4/18 14:11 UTC+08:00
**Refractive Index**	1.333	**Channel Width**	24 µsec (auto)
**Viscosity**	0.933 cP	**Temperature**	23 °C
**Particle Type**	Solid	**Intensity Set Point**	300 KHz
**Cell Type**	Square	**First Channel Used**	2

Analysis Results - Gaussian Distribution

	Intensity	Volume	Number
**Mean Diameter**	**201.29**	**135.87**	**55.06**
St.Dev.	95.61	64.54	26.15
CV %	47.50%	47.50%	47.50%
PI	0.23	0.23	0.23

Cumulative Results	Intensity	Volume	Number
25% of distribution <	130.38	88.03	37.94
50% of distribution <	179.67	121.30	50.86
75% of distribution <	247.59	167.14	69.22
80% of distribution <	268.07	180.96	74.80
90% of distribution <	330.42	223.04	91.83
99% of distribution <	542.70	366.33	150.02

**Run Time**	0:05:03
**Chi²**	37.911
**Baseline Adj.**	0.000
**Decay**	2.465
**Fit Error**	2.593
**Residual**	9.659
**Minimun Diameter**	10 nm
**Plot Size**	45
**Smoothing**	3
**Plot Range**	100

Printed:
2019/4/18 14:13 UTC+08:00 - zyy

Measured:
2019/4/18 14:11 UTC+08:00 - zyy
Approved:
2019/4/18 14:11 UTC+08:00 - zzw

Figure S7. The particle size and distribution of the cabazitaxel liposome (1)

Particle Size Analysis

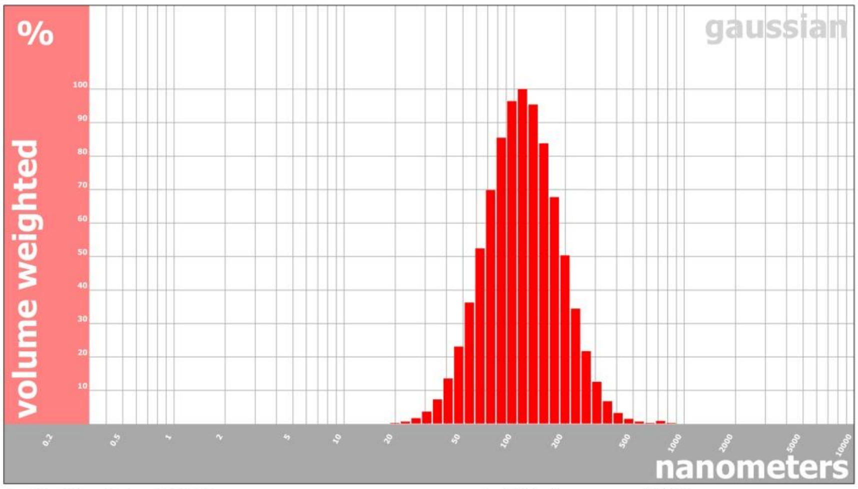

Particle Sizing System NICOMP **Serial Number** 1808303

cabazitaxel liposome - Lot: 2018111401

Custom Recipe

**Sample Date**	2019/4/18	**Measurement Date**	2019/4/18 14:21 UTC+08:00
**Refractive Index**	1.333	**Channel Width**	23 µsec (auto)
**Viscosity**	0.933 cP	**Temperature**	23 °C
**Particle Type**	Solid	**Intensity Set Point**	300 KHz
**Cell Type**	Square	**First Channel Used**	2

Analysis Results - Gaussian Distribution

	Intensity	Volume	Number
**Mean Diameter**	**198.88**	**125.56**	**48.24**
St.Dev.	99.24	62.66	24.07
CV %	49.90%	49.90%	49.90%
PI	0.25	0.25	0.25

**Cumulative Results**	**Intensity**	**Volume**	**Number**
25% of distribution <	125.35	79.21	34.21
50% of distribution <	175.50	110.85	45.54
75% of distribution <	245.71	155.18	62.26
80% of distribution <	267.08	168.67	67.41
90% of distribution <	332.63	210.05	83.30
99% of distribution <	559.93	353.55	138.71

**Run Time**	0:05:04
**Chi²**	26.843
**Baseline Adj.**	0.000
**Decay**	2.390
**Fit Error**	3.199
**Residual**	19.693
**Minimun Diameter**	10 nm
**Plot Size**	45
**Smoothing**	3
**Plot Range**	100

Printed:
2019/4/18 14:23 UTC+08:00 - zyy

Measured:
2019/4/18 14:21 UTC+08:00 - zyy
Approved:
2019/4/18 14:21 UTC+08:00 - zzw

Figure S8. The particle size and distribution of the cabazitaxel liposome (2)

Particle Size Analysis

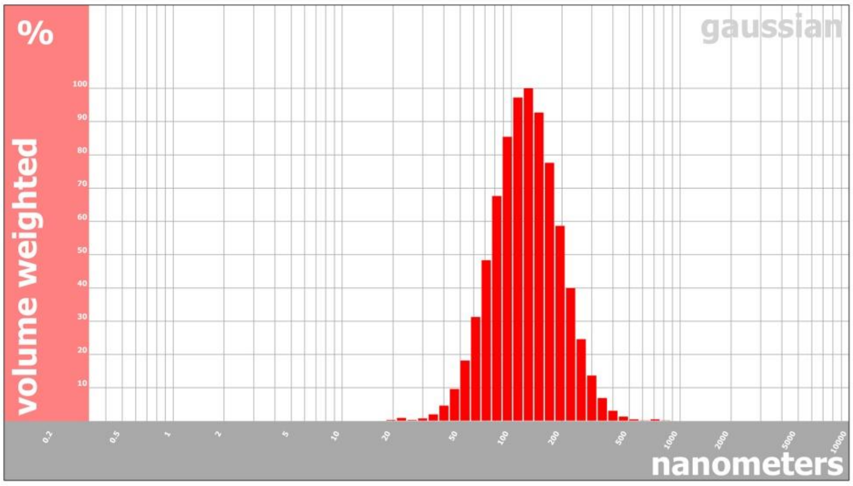

Particle Sizing System NICOMP **Serial Number** 1808303

cabazitaxel liposome - Lot: 2018111401

Custom Recipe

**Sample Date**	2019/4/18	**Measurement Date**	2019/4/18 14:30 UTC+08:00
**Refractive Index**	1.333	**Channel Width**	22 µsec (auto)
**Viscosity**	0.933 cP	**Temperature**	23 °C
**Particle Type**	Solid	**Intensity Set Point**	300 KHz
**Cell Type**	Square	**First Channel Used**	2

Analysis Results - Gaussian Distribution

	Intensity	Volume	Number
**Mean Diameter**	**194.65**	**135.21**	**61.01**
St.Dev.	87.40	60.71	27.39
CV %	44.90%	44.90%	44.90%
PI	0.20	0.20	0.20

Cumulative Results	Intensity	Volume	Number
25% of distribution <	129.93	90.26	41.70
50% of distribution <	175.88	122.18	55.84
75% of distribution <	238.08	165.38	75.23
80% of distribution <	256.63	178.27	81.04
90% of distribution <	312.66	217.18	98.58
99% of distribution <	499.55	347.00	157.19

**Run Time**	0:05:03
**Chi²**	12.689
**Baseline Adj.**	0.000
**Decay**	2.339
**Fit Error**	4.279
**Residual**	18.612
**Minimun Diameter**	10 nm
**Plot Size**	45
**Smoothing**	3
**Plot Range**	100

Printed:
2019/4/18 14:31 UTC+08:00 - zyy

Measured:
2019/4/18 14:30 UTC+08:00 - zyy
Approved:
2019/4/18 14:30 UTC+08:00 - zzw

Figure S9. The particle size and distribution of the cabazitaxel liposome (3)

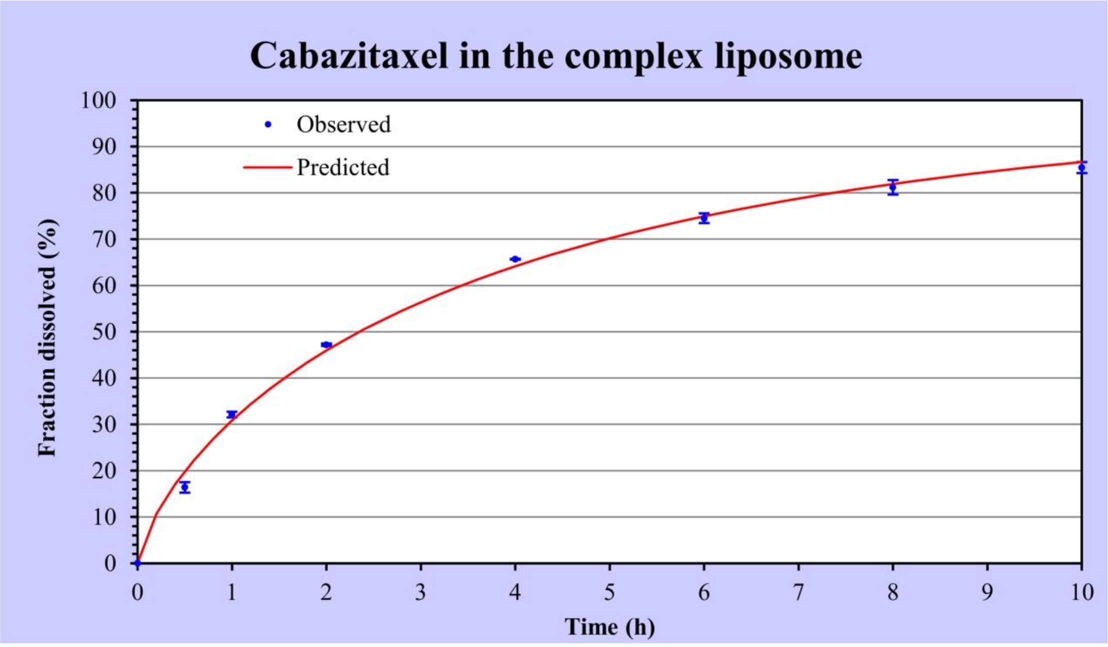

Figure S10. The release rate and fitting curve of cabazitaxel in the complex liposome

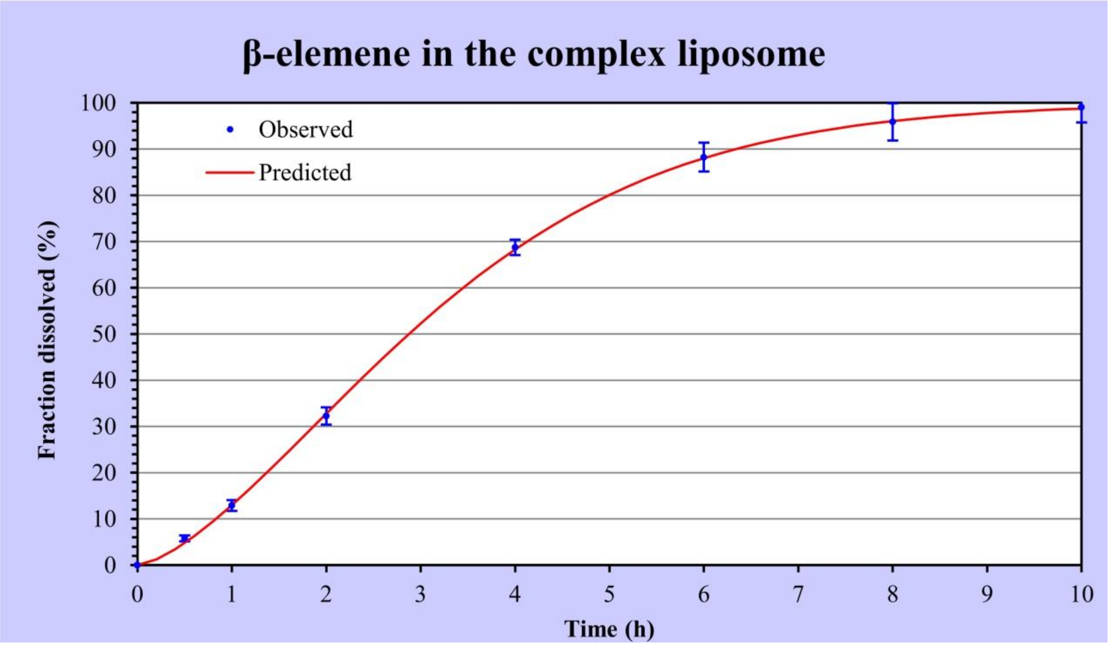

Figure S11. The release rate and fitting curve of β-elemene in the complex liposome

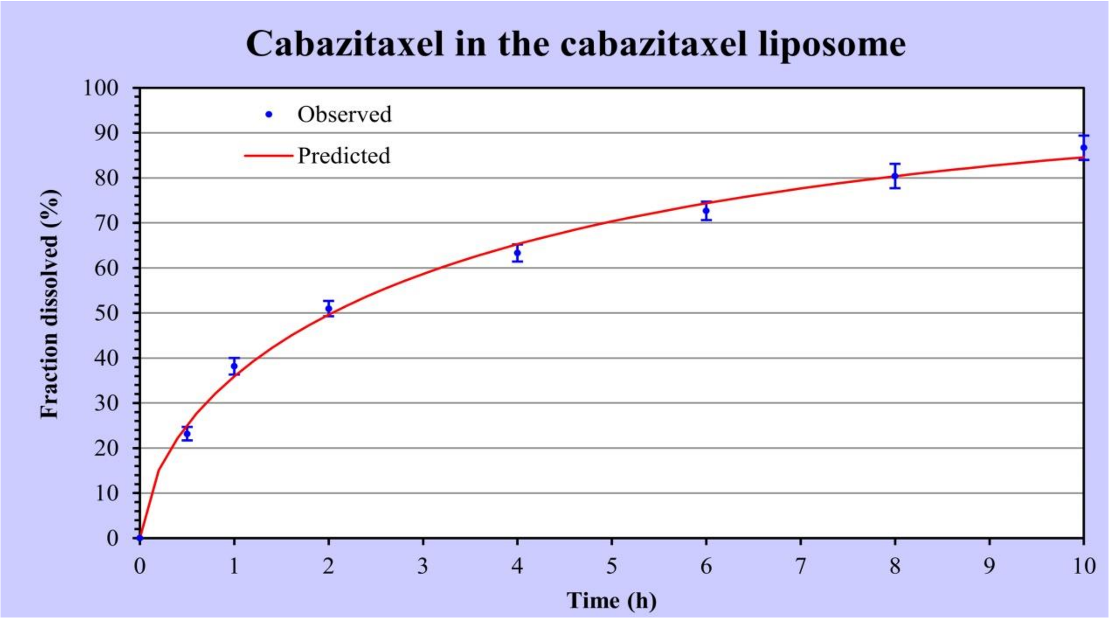

Figure S12. The release rate and fitting curve of cabazitaxel in the cabazitaxel liposome

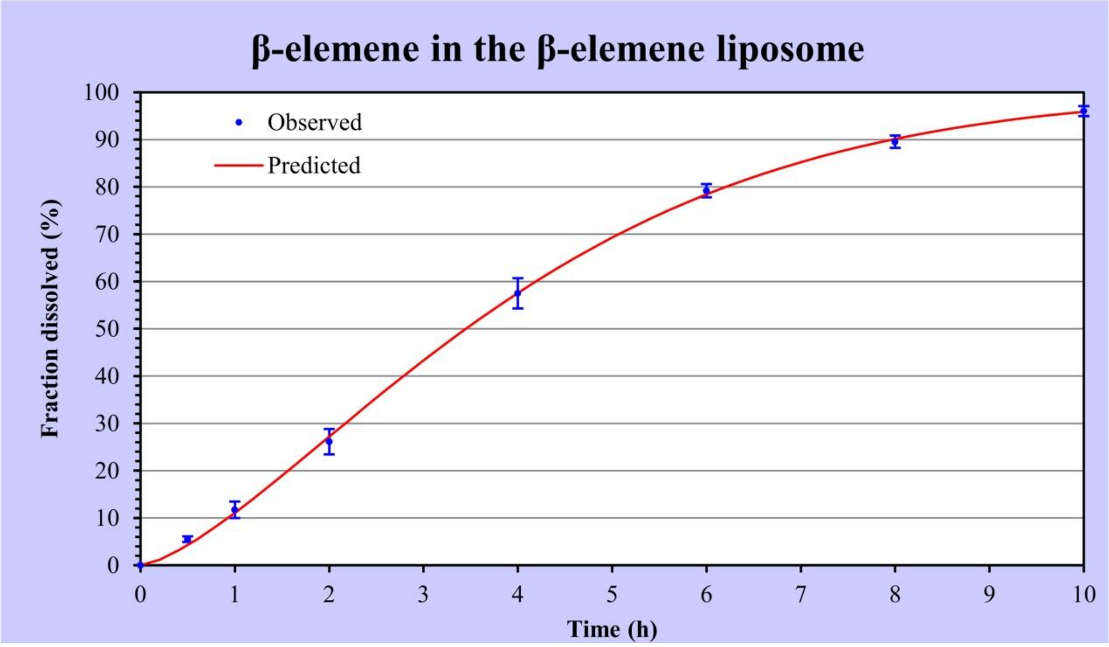

Figure S13. The release rate and fitting curve of β-elemene in the β-elemene liposome
